# Methodological advances in imaging intravital axonal transport

**DOI:** 10.12688/f1000research.10433.1

**Published:** 2017-03-01

**Authors:** James N. Sleigh, Alessio Vagnoni, Alison E. Twelvetrees, Giampietro Schiavo

**Affiliations:** 1 Sobell Department of Motor Neuroscience and Movement Disorders, Institute of Neurology, University College London, London, UK; 2 Division of Cell Biology, MRC Laboratory of Molecular Biology, Cambridge, UK; 3 Department of Basic and Clinical Neuroscience, Maurice Wohl Clinical Neuroscience Institute, Institute of Psychiatry, Psychology and Neuroscience, King's College London, London, UK

**Keywords:** axonal transport, intravital imaging, neurons

## Abstract

Axonal transport is the active process whereby neurons transport cargoes such as organelles and proteins anterogradely from the cell body to the axon terminal and retrogradely in the opposite direction. Bi-directional transport in axons is absolutely essential for the functioning and survival of neurons and appears to be negatively impacted by both aging and diseases of the nervous system, such as Alzheimer’s disease and amyotrophic lateral sclerosis. The movement of individual cargoes along axons has been studied *in vitro* in live neurons and tissue explants for a number of years; however, it is currently unclear as to whether these systems faithfully and consistently replicate the *in vivo *situation. A number of intravital techniques originally developed for studying diverse biological events have recently been adapted to monitor axonal transport in real-time in a range of live organisms and are providing novel insight into this dynamic process. Here, we highlight these methodological advances in intravital imaging of axonal transport, outlining key strengths and limitations while discussing findings, possible improvements, and outstanding questions.

## *In vivo* techniques are poised to provide novel insight into live axonal transport

Neurons are highly polarised, excitable cells with long, thin axons whose integrity requires specialised mechanisms to transport cargoes such as organelles (e.g. mitochondria) and molecules (e.g. proteins and RNA) in anterograde (from soma to axonal tips) and retrograde (from axonal tips to soma) directions ^1^. This bi-directional axonal transport is governed by the kinesin and cytoplasmic dynein motor proteins, respectively, and is essential for neuronal survival and function ^2^. Given the large distances over which these processes must occur, it is perhaps unsurprising that disturbances in axonal transport have been linked to both ageing and many severe nervous system diseases, including Alzheimer’s disease (AD) and amyotrophic lateral sclerosis (ALS) ^3– 5^. Emphasising the importance of efficient axonal transport to nervous system health, mutations in a number of motor proteins have been identified as causative in neuronal disorders ^1, 6, 7^.

**Figure 1.**
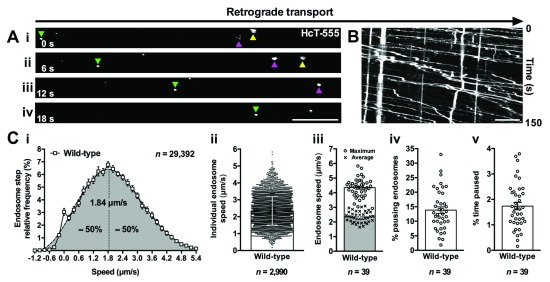
Imaging intravital axonal transport dynamics. ( **A**) The transport of individual fluorescent cargoes along axons can be assessed *in vivo*. This series of time-lapse confocal microscopy images (i-iv) depicts the retrograde movement (left to right) of the tetanus toxin binding domain (HcT-555) in sciatic nerve axons, as detailed in Gibbs *et al*. ^11^. Distinct signalling endosomes (e.g. coloured triangles) loaded with HcT-555 can be tracked across multiple images and transport assessed. ( **B**) Representative kymograph generated from fluorescently labelled signalling endosomes being transported in sciatic nerve axons. ( **C**) Numerous features of axonal transport kinetics can be assessed and plotted (see Box 3 also); for example, speed distribution curves of individual endosome steps (i), average endosome speeds (ii), mean endosome speeds per animal (iii), the percentage of endosomes that remain stationary for at least two consecutive frames (iv), and the percentage of time spent pausing (v). The data reported here were generated from 39 wild-type (C57BL/6) animals aged from 1 to over 13 months, which is a period when transport dynamics are known to remain stable ^71^. Scale bars (A–B) = 10 μm.

Individual cargoes have long been tracked in real-time in primary neuron and *ex vivo* tissue axons ^8– 10^; however, there is evidence to suggest that these artificial environments do not consistently reflect the *in vivo* situation ^11– 14^. Differences in transport dynamics, such as average speeds and pause frequencies ^11^, could be attributed to limitations inherent to these *in vitro* and *ex vivo* platforms. Cultured primary neurons lack the array of cells with which neurons normally physically and chemically interact *in situ*; for example, cultured motor neurons are not myelinated, do not contact target muscle cells, and lack a network of regulated excitatory and inhibitory input onto their cell bodies. Myelination ^15– 17^, target-derived signals ^18, 19^, and activity ^20– 23^ are all known to impact axonal transport, whereas non-cell autonomous and age-dependent disease mechanisms are difficult to accurately model *in vitro*. Mouse primary cultures are often derived from embryonic animals ^24, 25^ not currently analysed *in vivo*, which may also cause discrepancies, as could the current intrinsic variability of human induced pluripotent stem cell (hiPSC)-derived neuronal cultures ^26^. Furthermore, the artificially controlled *in vitro* environment could affect subcellular energy demands and transport kinetics ^27^, as might stress caused by axotomy and the continual growth of primary cultures. Intravital analysis of axonal transport of individual cargoes ( Figure 1) is therefore likely to provide more physiologically relevant insights into this dynamic process, albeit with its own pitfalls ( Table 1) ^28– 30^.

**Table 1.** The benefits and pitfalls of *in vivo* imaging of axonal transport compared with *in vitro* and *ex vivo* platforms.

Advantages	Disadvantages
Realistic physiological environment (e.g. chemical/ cellular interactions and energy demands)	Harder to study mechanism through experimental manipulation (lack of reductionist appeal)
Assessment not always restricted to particular time- points	Embryonic analysis is challenging and not currently possible in all models
Repeated (longitudinal) measurements across broad time scales	Distant subcellular comparisons are difficult owing to limited imaging fields (e.g. proximal versus distal axons)
Inherent variability of culturing processes and dissection artefacts avoided	Disease-relevant cells/tissues can be hard to access (e.g. mouse dopaminergic neurons)
Cellular stresses are limited (e.g. continual culture growth, dissection/dissociation)	Technically challenging in many instances

In this short review, we will highlight recent methodological advances and adaptations enabling the *in vivo* imaging of axonal transport across model organisms, specifically focusing on techniques that track the fast axonal transport of individual cargoes in real-time rather than transport *en masse*. We will outline strengths and weaknesses of the methods along with findings they have generated ( Box 1), highlight major outstanding questions ( Box 2), and discuss possible improvements and future directions for *in vivo* analysis.

Box 1. **Recent major insights into axonal transport provided by *in vivo* imaging**

- Consistent with sciatic nerve explant data ^5^, the percentage of motile mitochondria in the *Drosophila* marginal nerve declines with age, while the dynamic properties (run speed and length) remain unchanged ^45^
- Reduced levels of the dynein co-factor Lissencephaly-1 caused an increase in the percentage of motile mitochondria in the *Drosophila* marginal nerve ^45^
- Defective mitochondrial transport results in an increase of protein aggregation in *Drosophila* neurons; conversely, upregulating mitochondrial transport correlates with a delayed appearance of protein aggregates ^45^
- In zebrafish retinal ganglion cells (RGCs), disruption of Kif5A, a neuron-specific anterograde motor, resulted in increased frequency of retrograde mitochondrial transport but not synaptophysin-containing vesicles ^58^
- Zebrafish models of Charcot-Marie-Tooth disease type 2A ^59^ and Parkinson’s disease ^60^ showed mitochondrial motility defects in disease-relevant nerve cells
- During larval development (2–5 days post-fertilisation), the percentage motility of mitochondria and the ratio of anterograde to retrograde movements progressively decreased in zebrafish central nervous system (CNS) dopaminergic neurons, whereas run length increased, but speeds remained stable ^60^
- Amyotrophic lateral sclerosis (ALS) mice, but not spinal and bulbar muscular atrophy mice ^70^, display pre-symptomatic defects in the transport speeds of signalling endosomes in sciatic nerve axons ^67^; mitochondrial transport is also perturbed in both SOD and TDP-43 models of ALS ^67, 68^
- Retrograde axonal transport kinetics (speed, % pausing, and % time paused) of signalling endosomes in the sciatic nerve remain unchanged from 1 to over 13 months ^71^, which varies from the age-related transport deficiencies reported in different experimental settings at similar time points ^12, 89, 90^
- Bi-directional defects in the transport of both mitochondria and peroxisomes are detected in spinal cord axons of acute and chronic multiple sclerosis mouse models before major symptom onset ^77^
- In mouse RGCs, the number of moving mitochondria, but not run length, was decreased prior to cell death in a glaucoma model, whereas the duration and distance of mitochondrial transport were both diminished with age (23–25 months) ^12^
- The vast majority of mitochondria in neonatal and adult cortical pyramidal neurons remain stationary over periods of up to 20 minutes ^13, 14^

Box 2. **Outstanding questions that will benefit from advances in intravital imaging**

- What is the direct biological significance of altered cargo pausing and transport speeds?
- Are the defects in axonal transport observed in myriad neurological disease a cause of neuronal dysfunction or the consequences of a dysfunctional neuron?
  - Will therapeutics targeting axonal transport ameliorate symptoms of these diseases?
- Does ageing impact all neuronal and cargo subtypes equally?
  - What mechanisms underlie cargo-specific disturbances in transport versus global transport deficiencies?
- Why do mutations in mitochondrial ^91^ and motor proteins ^1^ often manifest in a nervous system-specific pathology?
  - Why are neurons particularly vulnerable to trafficking defects?
- What causes the axonal transport of distinct cargoes to be differentially affected by ageing and disease-associated mutations?

## Transport of diverse cargoes can be assessed in *Drosophila* wing sensory neurons over the lifespan of the animal

The sophisticated genetic toolboxes of *Caenorhabditis elegans* and *Drosophila melanogaster* allow the specific targeting of fluorescent proteins to vesicles and organelles such as mitochondria. When coupled with the ever-expanding repertoire of neurological disease-relevant worm and fly models ^31– 34^, these reporter lines provide a powerful system for analysing the axonal transport of assorted cargoes in both ageing and disease ^35– 40^. Transport studies in *Drosophila* have largely been performed in filleted larval preparations rather than adult flies, limiting the time period over which analyses can be performed and the developmental stage of the neurons under investigation. Third instar larvae are typically dissected for imaging of fluorescent cargoes predominantly in motor axons ^41, 42^. Alternatively, microfluidic devices that physically immobilise intact *Drosophila* larvae afford a non-invasive approach to image axonal transport ^43, 44^.

**Figure 2.**
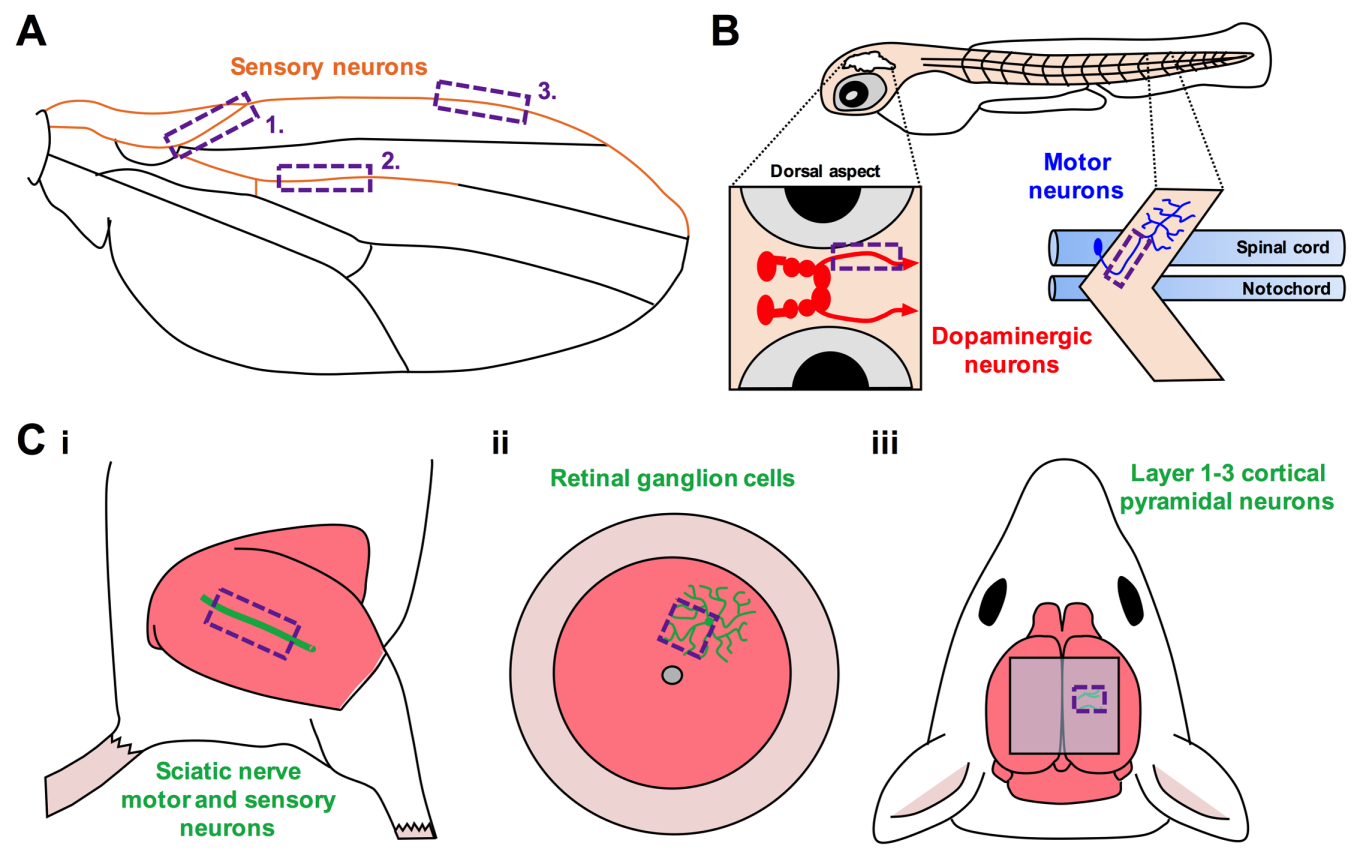
Developments in intravital imaging of axonal transport. ( **A**– **C**) Recent technical advances have permitted the assessment of axonal cargo dynamics in a range of neuron types across different live model organisms. In the past few years, organelles have been tracked for the first time in sensory neurons of the adult *Drosophila* wing (orange, **A**) ^45, 46^ and larval zebrafish retinal ganglion cells ^58^, central nervous system (CNS) dopaminergic neurons (red, **B**), and middle primary (MiP) motor neurons (blue, **B**) ^59, 60^. In the mouse, a more experimentally challenging animal model because of its non-translucency, *in vivo* transport was assessed in (i) motor and sensory sciatic nerves ^11, 68, 71, 72^, axons of the spinal cord and dorsal roots ^77^, (ii) retinal ganglion cells ^12^, and (iii) distal layer 1–3 cortical pyramidal neurons ^13, 14^ ( **C**). The purple, dashed-line boxes indicate approximate imaging regions.

A novel technique to analyse transport dynamics in sensory axons of the *Drosophila* wing has been developed ( Figure 2A), which permits the assessment of axonal trafficking throughout the lifespan of adult flies ^45, 46^. The marginal nerve found on the anterior edge of fly wings consists of chemosensory and mechanosensory neurons ^47, 48^, the cell bodies of which are connected by short dendrites to wing bristles, while their axons bundle together and project to the central nervous system (CNS) ^45^. Given the translucency of the wing and the accessibility of the marginal nerve, rapid and non-invasive light microscopy can be performed on different wing regions of flies expressing fluorescently tagged proteins specifically in neurons. Motivated by previous work in which the same nerve was used to evaluate *in vivo* responses to neuronal injury ^49, 50^, the GAL4-UAS system was implemented to visualise mitochondria and dense core vesicle (DCV) transport dynamics in flies up to 30 days old (their natural lifespan being ≈50 days in the laboratory) ^45^. Flies showed an early peak in the number of bi-directional moving mitochondria during early adulthood and subsequent decline with age linked to misfolded axonal protein accumulations; however, the dynamic properties of the moving mitochondria did not change with time. Intriguingly, reduced levels of the dynein co-factor Lissencephaly-1 (Lis1) caused a doubling in the number of motile mitochondria across time-points (without increasing their number) and reduced the age-associated build-up of protein in the axon ^45^. Contrasting with mitochondria, the percentage of motile DCVs remained steady across ages in wild-type flies and was unaffected by reduced Lis1 levels, which is indicative of an organelle-specific perturbation rather than a global transport defect. Although the *Drosophila* marginal nerve cannot be used for whole-mount staining or large-scale biochemistry and has no direct counterpart in humans, its simple preparation permits quick and non-invasive analysis of anterograde and retrograde axonal transport in sensory nerves of adult flies. Without constraints on fly age, extended experimental time-points can be incorporated, facilitating the study of ageing and neurodegeneration in *Drosophila*.

## Assessment of cargo motility in axons from an array of zebrafish neuronal subtypes

As a genetic model, the zebrafish ( *Danio rerio*) possesses many of the advantages of the invertebrate species, such as short generation time and lower maintenance costs, with the added benefit of being a vertebrate with myelinated axon tracts ^51^. Also, like worms and flies, zebrafish are highly genetically tractable, with an array of reporter lines expressing fluorescently tagged proteins in specified organelles in subsets of cells. Zebrafish larvae are translucent, which obviates the need for invasive imaging techniques and allows repeated, longitudinal *in vivo* measurements throughout development. Several studies have probed cargo movement in the axons of anaesthetised zebrafish, the first of which assessed mitochondrial dynamics in sensory nerves called Rohon-Beard (RB) neurons found in the tail tip ^52^. These transgenic “MitoFish” were created using the GAL4-UAS system to specifically express fluorescent proteins in the mitochondria of single RB neurons. A number of other groups have created similar fluorescent fish to assess the dynamics of RB mitochondria ^53^ and endosomes ^54, 55^, as well as lysosomes in mechanosensory axons ^56, 57^.

Until recently, the bulk of *in vivo* zebrafish transport analysis was conducted in sensory nerves; however, a number of groups have capitalised on the translucency and genetics of the fish and expanded the arsenal of neuronal types amenable to imaging. To assess the visual system, transgenic fish expressing fluorescent protein in either the synaptic vesicles or the mitochondria of retinal ganglion cells (RGCs) were created ^58^. Disruption of the neuron-specific anterograde motor Kif5A increased the number of small motile synaptophysin-containing vesicles at early developmental stages without altering the ratio of anterograde to retrograde transport. In contrast, the percentage of mobile mitochondria was unaffected in *Kif5 ^–/–^* animals, but mitochondria were transported more frequently in the retrograde direction, which likely causes the observed depletion of axonal mitochondria. Similar to the above *Drosophila* study ^45^, these experiments once again highlight that transport of distinct organelles can be differentially impacted depending on the type and stoichiometry of the motor proteins driving their movement. The results also emphasise the importance of measuring multiple transport parameters ( Box 3), as different conclusions would have been reported if just the percentage of motile mitochondria was assessed. In addition to RGCs, new transgenic lines have been generated to assess mitochondrial dynamics in middle primary (MiP) motor neurons of the spinal cord ^59^ and CNS dopaminergic neurons ^60^ ( Figure 2B). These fish were used to show that models of Charcot-Marie-Tooth disease type 2A (CMT2A) ^59^ and Parkinson’s disease (PD) ^60^ display perturbations in mitochondrial motility in disease-relevant neuronal subtypes; CMT2A motor nerves had a reduced percentage of motile mitochondria with unchanged speeds, while PD dopaminergic neurons displayed multiple transport defects dependent on the dose of toxin (MPP ^+^) used to model the disease. The percentage of motile mitochondria and the ratio of anterograde to retrograde movements decreased during larval development (2–5 days post-fertilisation) in wild-type dopaminergic neurons, while speeds remained stable and run lengths increased ^60^. Given the assortment of neurons now available for imaging, zebrafish provide an exciting platform for the *in vivo* analysis of axonal transport during development, with the caveat that once zebrafish reach adulthood, they become opaque, abrogating their utility for post-larval analyses.

Box 3. **Axonal transport analysis.**

A multitude of subtly different and sometimes co-dependent axonal transport parameters may be measured. Some features, such as the ratio of anterograde to retrograde movements, cannot be assessed for certain cargoes, e.g. signalling endosomes, which are transported only from the periphery to the cell soma. Cargo type should thus be considered and the aims of each individual experiment carefully determined before selecting from the following analysis options, which may also be subdivided into anterograde, retrograde, bi-directional, and combined categories:

- **Speed**
  - Frequency of frame-to-frame speeds ( Figure 1Ci) ^11, 67, 70, 71^
  - Individual cargo average velocities ( Figure 1Cii) ^71^
  - Average ^52, 56, 71, 92^ and maximum cargo speeds ( Figure 1Ciii) ^71^
  - Immobile cargoes can be either included or omitted, and analysed separately ^59^, while movement-only speeds (i.e. uninterrupted runs or constant-velocity segments) can be determined ^52, 92^
- **Motility**
  - Percentage ^14, 35, 45^ and number ^20, 52, 56, 92^ of motile cargoes in a given time (also called flux)
  - Percentage of time motile cargoes are moving ^12, 60^
  - Average ^56^ and longest ^45^ run distances (run length)
  - Run duration ^12, 13, 39^
- **Pausing**
  - Percentage of cargoes that pause ( Figure 1Civ) ^67, 71^
  - Percentage ^21, 71^ and length ^52, 77, 92^ of time that motile cargoes remain stationary ( Figure 1Cv)
  - Pause frequency ^52, 92^
  - Percentage ^13, 58^ and number ^20^ of cargoes that remain stationary
- **Anterograde, retrograde, and bi-directional**
  - Ratio of anterograde to retrograde movements ^58^/net direction of transport ^35^
  - Percentage of time spent anterogradely moving/stationary/retrogradely moving ^36^
  - Frequency and percentage of cargoes that show reversals in transport direction ^35^
  - Percentage of cargoes that oscillate ^36^ or remain uni-directional ^60^

Microscope settings can also impact axonal transport results, so care must be taken when making cross-study comparisons. Furthermore, whether recordings will be manually or automatically tracked must also be taken into account. The following should therefore be contemplated:

- **Frame rate:** there is always a trade-off between sampling frequency and specimen integrity ^93, 94^. For example, a low-frequency frame rate (less than 1 Hz) could miss brief pauses, resulting in the recording of slower “moving” speeds for individual cargoes and fewer pauses. Rapid frame rates may provide more accurate information but must be offset against how rapidly a sample bleaches, potential phototoxic changes to specimen physiology, and the signal-to-noise ratio. Frame rate also directly impacts the labour required for analysis if manual tracking of cargoes is carried out.
- **Imaging time:** the longer an axon is imaged, the greater the chance that stationary organelles, particularly mitochondria, will become motile. The impact of phototoxicity should also be considered. Imaging over several orders of magnitude can circumvent this problem: for example, imaging at 2 Hz for 1 minute followed by 0.2 Hz for 10 minutes ^95^.

## Peripheral and central nerve transport dynamics can both be assessed in mice

Intravital imaging techniques have been developed in mice to study a range of biological processes *in vivo*, including the response to spinal cord injury ^61^, retinal degeneration ^62^, and cortical function and development ^63, 64^. Similar to the animal models mentioned above, experimentalists working with mice can use an extended library of transgenic fluorescent reporter strains facilitating live imaging ^28, 65^. Consequently, there has been a recent flurry of publications adapting these *in vivo* techniques for the analysis of axonal transport in live mice.

Among these, transgenic mice selectively expressing fluorescent proteins in neuronal mitochondria called “MitoMice” were generated by the Lichtman laboratory in 2007 ^66^. Mitochondria are labelled throughout most of the “MitoMouse” nervous system, permitting *in vivo* analysis across a broad spectrum of neuronal subtypes provided they can be accessed in live animals. In this publication, *in vivo* axonal transport of individual organelles was imaged for the first time in a live mammal ^66^. Mitochondrial kinetics were analysed in single motor and sensory axons of surgically exposed sciatic nerves using time-lapse confocal microscopy ( Figure 2Ci).

Adaptations of this technique have since been developed and expounded upon by a number of different laboratories. The Schiavo group crossed the “MitoMouse” with the SOD1 ^G93A^ mouse model of ALS and showed that mitochondrial transport speeds are pre-symptomatically impaired in sciatic nerve axons, which is one of the first observable deficiencies in this disease model ^67^. Another laboratory confirmed this result and expanded it to the TDP-43 ^A315T^ mouse model of ALS ^68^. Using a fluorescently tagged atoxic binding fragment of tetanus neurotoxin (H _C_T), the dynamics of a second type of organelle, the signalling endosome, were assessed in the sciatic nerve of SOD1 ^G93A^ mice ( Figure 1) ^67^. Fluorescent H _C_T was injected under anaesthesia into the gastrocnemius and tibialis anterior muscles of the lower leg, where it binds to nidogen receptor proteins of the basement membrane before internalisation at the neuromuscular junction ^69^. Once in the nerve terminal, the toxin hijacks the retrogradely transported signalling endosomes, which can be tracked in sciatic nerve axons *in vivo* ^11^. Signalling endosome movement was also shown to be impaired ^67^, suggestive of a generalised transport defect in SOD mutant mice. This is most likely caused by global changes in general transport machinery, e.g. the microtubule network, as opposed to disruption of multiple cargo-specific pathways. Confirming that defective retrograde transport of signalling endosomes is not a general read out of an impaired or aged nervous system, in separate studies spinal and bulbar muscular atrophy mice ^70^ and wild-type animals aged to over 13 months ^71^ were both shown to have normal endosome transport dynamics. A minor drawback of these studies is that the identity of sciatic nerve motor and sensory axons cannot be readily differentiated. Nonetheless, there is the possibility of injecting H _C_T into the footpad to target nociceptive sensory neurons or injecting a fluorescently labelled p75 ^NTR^ neurotrophin antibody into muscle, which is mainly taken up by sensory nerves ^11, 67^. Moreover, crossing of disease models with transgenic mice selectively expressing fluorescent proteins in motor axons (e.g. using the Hb9/Mnx1 promoter) could also help to overcome this issue. Alternatively, the mainly motor femoral nerve ^72^ or primarily sensory sural ^72^ or saphenous nerves ^20^ could be imaged either in fluorescent reporter strains or by altering the H _C_T injection site to ensure fluorescent signalling endosome transport in the appropriate nerve. However, these alternative peripheral nerves are more technically challenging to image on an inverted microscope because of their anatomical distribution and size. Indeed, the sciatic nerve is a large, superficial collection of peripheral nerve axons, in which the dynamics of various cargoes can be imaged, with the useful possibility for longitudinal analysis in the same animal ^66, 72^.

In contrast, the mouse CNS is inherently more difficult than the relatively accessible peripheral nervous system to image directly; nevertheless, axonal transport has now been successfully tracked in a number of CNS neurons. Axons within the spinal cord can be surgically exposed by dorsal laminectomy and imaged across time and several spinal segments *in vivo* ^61, 73– 75^, permitting longitudinal and location-specific comparisons. Mice expressing fluorescent proteins in only a small percentage of sensory neurons have been used to assess axonal degeneration caused by spinal cord lesion in individual large, myelinated sensory axons found in the dorsal aspect of the spinal cord ^61^. Numerous synthetic vital dyes that label structures such as myelin and microglia can also be used to aid *in vivo* analysis of the spinal cord ^76^. This technique was recently implemented to assess axonal transport in acute and chronic mouse models of multiple sclerosis (MS) ^77^. Crossing these strains with “MitoMice” and specifically generated “Thy1-PeroxiYFP” mice, both mitochondria and peroxisomes, respectively, were imaged in spinal cord and dorsal root axons. Pervasive defects in anterograde and retrograde transport of both cargo types were observed in MS mouse spinal cord axons before the onset of additional deficiencies, suggesting that axonal transport defects represent an early and important pathological sign in this disease ^77^. These defects were not seen in dorsal root axons (sensory nerves), suggesting that subcellular location (i.e. proximity to the soma) has a bearing on axonal transport defects. However, the sensory identity of the axons imaged in the spinal cord was only presumed (owing to their dorsal location) and not experimentally confirmed, so the potential site-specific transport issue could instead be a product of neuron subtype (e.g. motor versus sensory).

Multi-photon microscopy has also been used to assess mitochondrial transport dynamics in the axons of RGCs of anaesthetised “MitoMice” ( Figure 2Cii) ^12^. RGC axons extend into the nerve fibre layer of the eye, parallel to the ocular surface of the sclera, permitting the visualisation of organelles after subtle opening of the surrounding skin and conjunctiva. The number of moving mitochondria, but not their run length, was diminished before RGC death in a model of glaucoma, whereas in aged mice (23–25 months), the duration and distance of mitochondrial movement were both diminished ^12^. Interestingly, counter to results from retinal explants in the same study, this method showed that mitochondrial transport is highly dynamic in the mouse CNS *in vivo* ^12^. Unfortunately, as fluorescence microscopy could impact the activity of light-sensitive retinal neurons, this observation may be confounded by artefactual alterations in mitochondrial dynamics (i.e. caused by increased activity ^20^). Furthermore, this technique is restricted to albino strains because uveal pigmentation prevents imaging, and is more technically challenging with gaseous anaesthetics because of face access requirement. Nonetheless, *in vivo* comparisons of ** organelle transport in proximal and distal regions of RGC axons allow for repeated, longitudinal analyses.

Finally, a technique developed to image distal layer 1–3 pyramidal neurons of the cortex has been independently adapted by two groups to assess axonal transport through a surgically fitted cranial window in both anaesthetised and awake mice ( Figure 2Ciii) ^13, 14^. Rather than using transgenic fluorescent strains, plasmids were unilaterally electroporated into embryos *in utero* to target the expression of fluorescent proteins to both the mitochondria and the cytoplasm ^13^ or membranes ^14^ of cortical progenitor cells (the latter to aid the identification of successfully transfected neurons). The process of electroporation restricts the fluorescence to a small proportion of axons, facilitating the assessment of individual collaterals. Similar to previous *in vivo* reports from zebrafish RB sensory ^52^ and CNS dopaminergic ^60^ neurons and mouse sciatic nerve axons ^66^, the percentage of immobile mitochondria in cortical axons was very high over short time periods: >99% in 2 minutes in both pups (P10–13) and adults (P70–120) ^14^ and ≈90% at P10–12 over 10–20 minutes ^13^. The discrepancy between these two studies could reflect the different but overlapping cortical layers that were imaged; however, it is more likely caused by the 5- to 10-fold difference in imaging periods. Indeed, over 95% of mitochondria were reported as stationary in mature cortical neurons in culture when imaged for 30 minutes, which drops to ≈75% using a 12-hour imaging window ^13^. This highlights a key problem in the study of axonal transport: to track individual cargo movements, rapid frame rates are required, but biologically relevant transport events may occur separated by much longer periods (minutes to hours), which is particularly challenging for *in vivo* imaging ( Box 3). Thus, although surgically intensive and technically demanding, this method of imaging cortical collaterals allows repeated, longitudinal analyses of CNS neurons of the brain in live, non-anaesthetised mice.

## Concluding remarks

The active transport of organelles and molecules along axons is critical to neuronal health, function, and survival. Deficiencies in this process appear to be intricately linked to ageing and neurodegeneration, but whether they play a causal role or are simply a consequence of a pathologically affected tissue remains to be fully elucidated in each setting ( Box 2). There are numerous possibly conflicting data reported on the dynamics of axonal cargoes. These differences could be due to several reasons, including experimental setting and parameters, neuronal subtype, cargo type, time-point, axonal location (i.e. distal versus proximal ^78^), and neuronal morphology (e.g. proximity to axonal arbor branches ^52^). With recent developments discussed here, we are beginning to acquire a diverse and very powerful arsenal of *in vivo* experimental systems across model organisms that will greatly enrich our understanding of transport. It is now vital to implement these intravital methods to tackle the questions of when (age), where (neuron category and subcellular location), what (cargo type), and how axonal transport deficiencies manifest in neuronal dysfunction.

Progress in imaging axonal transport *in vivo* has challenges in common with all *in vivo* imaging experiments (e.g. limited transparency of tissue, restricted imaging depth, and phototoxicity) as well as formidable problems unique to the phenomena being studied: namely, relevant scales of measurement that span several orders of magnitude, both in time and distance. The difficulty in labelling cargoes specifically and with a labelling density allowing a sufficient signal-to-noise ratio for transport analysis is highlighted by the small number of axonal cargoes currently being studied, which are almost exclusively membranous organelles. Using bright, photoactivatable fluorophores is a particularly helpful strategy for studying transport, as it allows the tracking of subpopulations of cargo otherwise too dense to study individually or sparse cargoes over long time periods ^13^. However, there is an urgent need to adopt new labelling strategies and fluorescent reporters to understand the behaviour of non-membrane-bound organelles, particularly in the field of RNA transport. Much of what has been learnt about organelle transport is garnered from experiments on mitochondria ^30^; however, their movement within axons, which is characteristically interspersed by long pauses and thus perhaps more characteristic of slow axonal transport, is not reflective of all cargo types. This is perhaps because they have distinct axonal roles, rely on specific subsets of adaptor and motor proteins ^2^, and are unique, highly dynamic, network-forming organelles. To have a thorough and informed understanding of axonal transport in health and disease, it is therefore of paramount importance that multiple cargo types are analysed.

Most of the methods discussed here are adaptations of intravital techniques initially developed to study other biological processes. It is therefore likely that imaging of additional neuronal subtypes or subcellular locations ^79– 86^ could be incorporated into these analyses in order to provide a more global assessment of *in vivo* axonal transport in health and disease. Moreover, as imaging techniques become more sophisticated, allowing high-speed, multi-channel acquisition at greater tissue depths ^87, 88^, we will be able to simultaneously monitor the dynamics of different organelles in their native environment and reliably assess the transport of organelles, such as RNA granules, for which similar robust protocols are currently lacking.
